# Investigation of antimicrobial resistance patterns and molecular typing of *Pseudomonas aeruginosa* isolates among Coronavirus disease-19 patients

**DOI:** 10.1186/s12866-023-02825-w

**Published:** 2023-03-29

**Authors:** Somaye Shiralizadeh, Fariba Keramat, Seyyed Hamid Hashemi, Mohammad Mehdi Majzoobi, Masoud Azimzadeh, Mohammad Sina Alikhani, Pezhman Karami, Zahra Rahimi, Mohammad Yousef Alikhani

**Affiliations:** 1 Department of Microbiology, Faculty of Medicine, Hamadan University of Medical Sciences, Hamadan, IR Iran; 2 Department of Infectious Diseases, Faculty of Medicine, Hamadan University of Medical Sciences, Hamadan, IR Iran; 3 Infectious Disease Research Center, Hamadan University of Medical Sciences, Hamadan, IR Iran; 4 Students Research Committee, Hamadan University of Medical Sciences, Hamadan, IR Iran

**Keywords:** COVID-19, Co-infection, *Pseudomonas aeruginosa*, Antimicrobial resistance, Biofilm formation, MLVA

## Abstract

**Background:**

*Pseudomonas aeruginosa* is a common co-infecting pathogen recognized among COVID-19 patients. We aimed to investigate the antimicrobial resistance patterns and molecular typing of *Pseudomonas aeruginosa* isolates among Coronavirus disease-19 patients.

**Methods:**

Between December 2020 and July 2021, 15 *Pseudomonas aeruginosa* were isolated from COVID-19 patients in the intensive care unit at Sina Hospital in Hamadan, west of Iran. The antimicrobial resistance of the isolates was determined by disk diffusion and broth microdilution methods. The double-disk synergy method, Modified Hodge test, and polymerase chain reaction were utilized to detect *Pseudomonas aeruginosa* extended spectrum beta-lactamase and carbapenemase producers. Microtiter plate assay was performed to evaluate the biofilm formation ability of the isolates. The isolates phylogenetic relatedness was revealed using the multilocus variable-number tandem-repeat analysis method.

**Results:**

The results showed *Pseudomonas aeruginosa* isolates had the most elevated resistance to imipenem (93.3%), trimethoprim-sulfamethoxazole (93.3%), ceftriaxone (80%), ceftazidime (80%), gentamicin (60%), levofloxacin (60%), ciprofloxacin (60%), and cefepime (60%). In the broth microdilution method, 100%, 100%, 20%, and 13.3% of isolates showed resistance to imipenem, meropenem, polymyxin B, and colistin, respectively. Ten (66.6%) isolates were identified as multiple drug resistance. Carbapenemase enzymes and extended spectrum beta-lactamases were identified in 66.6% and 20% of the isolates, respectively and the biofilm formation was detected in 100% of the isolates. The *bla*_OXA-48_, *bla*_TEM_, *bla*_IMP_, *bla*_SPM_, *bla*_PER_, *bla*_VEB_, *bla*_NDM_, *bla*_SHV_, and *bla*_CTX-M_ genes were detected in 100%, 86.6%, 86.6%, 40%, 20%, 20%, 13.3%, 6.6%, and 6.6% of the isolates, respectively. The *bla*_*VIM*_*, bla*_*GIM*_*, bla*_*GES*_*, and bla*_*MCR-1*_ genes were not identified in any of the isolates. The MLVA typing technique showed 11 types and seven main clusters and most isolates belong to cluster I, V and VII.

**Conclusion:**

Due to the high rate of antimicrobial resistance, as well as the genetic diversity of *Pseudomonas aeruginosa* isolates from COVID-19 patients, it is indispensable to monitor the antimicrobial resistance pattern and epidemiology of the isolates on a regular basis.

## Background

The Severe Acute Respiratory Syndrome Coronavirus 2 (SARS-CoV-2) is bringing forth Coronavirus Disease 2019 (COVID-19), giving health systems and clinicians a difficult medical challenge [1]. Hospitalized patients, specifically COVID-19 patients, receive antibiotics without sufficient scientific evidence and clinical experience. Although co-infections between viruses and bacteria can have severe consequences, not much information is available on the coinfection of bacteria with SARS-CoV-2 [2]. COVID-19 patients are affected by a number of bacteria, including *P. aeruginosa* [3]. *P. aeruginosa* is an opportunistic organism that causes nosocomial infections (such as pneumonia, urinary tract infections, bloodstream infections, surgical site infections and burn wound infections), and infections in immunocompromised patients (especially neutropenia and malignancy blood) as well as one of the leading causes of disability and death for patients with cystic fibrosis (CF) and non-CF bronchiectasis [4]. In *P. aeruginosa*, pathogenesis is conducted by adhesions (flagella and type IV pilli), secreted toxins, proteases, effector proteins (such as ExoS, ExoT, ExoU, and ExoY produced by the type III secretion system) and pigments that induce adhesion, regulate or interrupt host cell pathways, and interact with the external matrix. Its ability to cause severe infections is also enhanced by quorum sensing and biofilm formation [5]. Multidrug resistance has increased worldwide, which is considered a threat to public health. Several recent studies have reported the emergence of multidrug-resistant bacterial pathogens from different origins, which increases the need for proper use of antibiotics. In addition, the routine use of antimicrobial susceptibility testing is necessary to detect the antibiotic of choice as well as to screen for emerging MDR strains [6–8].

The rising prevalence of nosocomial infections caused by MDR *P. aeruginosa* is related to a considerable increase in morbidity and fatality due to limitations in the selection of appropriate antibiotics [9, 10]. The selective treatment of *P. aeruginosa*-infections is the use of beta-lactam antibiotics [11]. Resistance to the mentioned antibiotics is extending and is done by diverse resistance mechanisms, including the breakdown of antibiotics by β-lactamase enzymes such as Extended-spectrum-β-lactamases (ESBLs), the excretion of antibiotics by efflux pumps, and the reduction of drug absorption [12, 13]. The biofilm formation by *P. aeruginosa* is responsible for hospital-acquired infections and contributes to persistent colonization in tissues. Biofilms protect bacteria from antibiotics and host immune reactions and contribute to the interchange of resistance genes among microorganisms [14, 15]. Besides routine susceptibility tests to antimicrobial agents, typing pathogenic microorganisms isolated from hospitalized patients, particularly patients with COVID-19, can provide helpful information for physicians. Several genotyping procedures have been applied to investigate the epidemiology and genetic relatedness of *P. aeruginosa* isolates in the primary phase of infection. Due to the faster, less complicated, and inexpensive Multilocus Variable Number Tandem Repeat Analysis (MLVA) technique, it has become increasingly popular to characterize microorganisms [16, 17]. The foundation of the MLVA method is the proliferation of sequences containing a variable number of tandem repeats (VNTRs) in particular loci on microorganisms' genomes. The difference in the numeral of repetitions from a VNTR allows the strain's discrimination [18]. Therefore, the purpose of this study was to investigate the antibiotic resistance patterns and molecular typing of *Pseudomonas aeruginosa* strains that were isolated from patients with Covid-19.

## Methods

### Isolation and identification of *P. aeruginosa*

From December 2020 to July 2021, fifteen *P. aeruginosa* were isolated from COVID-19 patients in the intensive care unit (ICU) at Sina Hospital in Hamadan, west of Iran. The collected *P. aeruginosa* isolates were cultured on blood agar, MacConkey agar and cetrimide agar (Merck, Germany). After incubation for 24 h at 37 ºC, the grown colonies were examined in terms of morphology and pigment production and gram staining. Lactose negative bacteria were purified to perform confirmatory tests. The growth at 42 ºC, oxidase test, TSI, urease, simon citrate and oxidative/fermentative media (Merck, Germany) were used to confirm the *P. aeruginosa* isolates [19, 20].

### Antimicrobial susceptibility testing

The disc diffusion method was accomplished using guided by the Clinical and Laboratory Standards Institute (CLSI) criteria [21]. Briefly, a standardized inoculum was cultured onto the surface of Mueller–Hinton (MH) agar (Merck, Germany) and antibiotic disks were placed on the surface of the agar, and the size of the zone of inhibition around the disk was measured after overnight incubation at 37 °C. The following antibiotic disks (Condalab, Spain) were used: carbapenems (imipenem 10 μg), cephalosporins (cefepime 30 μg, ceftazidime 30 μg, and ceftriaxone 30 μg), fluoroquinolones (ciprofloxacin 5 μg, and levofloxacin 5 μg), aminoglycosides (gentamicin 10 μg), and trimethoprim-sulfamethoxazole (1.25—23.75 μg). *P. aeruginosa* ATCC 27,853 was used as control positive organism. The MDR, extensively drug-resistant (XDR), and pandrug-resistant (PDR) isolates categorized based on criteria defined by Magiorakos et al. [22]. When performing routine antimicrobial susceptibility testing on bacterial isolates in clinical microbiology laboratories, the limited number of agents generally tested will result in many MDR bacteria being categorized as ‘possible XDR’ or possible PDR’ [22].

The broth microdilution method was conducted to determine the minimum inhibitory concentration (MIC) of the imipenem, meropenem, colistin, and polymyxin B (Sigma-Aldrich/USA) and interpreted as CLSI guidelines [21]. Briefly, the bacterial isolates were inoculated into a MH broth in the presence of different concentrations of an antimicrobial agent and the growth of bacteria was assessed after incubation (16–20 h) at 37 °C and the MIC value was determined.

### Phenotypic ESBLs examination

Combination Disk Test (CDT) was applied to identify ESBL-producing isolates using ceftazidime, ceftazidime-clavulanic acid, cefotaxime, and cefotaxime-clavulanic acid disks (Oxoid, UK). After incubation, the difference of > 5 mm in zones of growth inhibition for a disk with clavulanic acid compared to a disc without clavulanic acid is indicative of the presence of ESBLs [23].

### Phenotypic carbapenemase examination

The Modified Hodge test (MHT) was conducted according to CLSI guidelines [21]. Briefly, a suspension of the indicator organism of *Escherichia coli* ATCC 25,922 was prepared and lawn cultured. The meropenem disk (Condalab, Spain) was put in the center of the plate. After that, a colony of the test organisms was inoculated onto the plate and incubated at 37 °C for 18 h. The cloverleaf-like structure indicated the production of carbapenemase.

### Biofilm assay

Microtiter plate (MTP) assay evaluated biofilm formation as described previously [24]. Briefly, the isolates were cultured in Luria–Bertani broth (LB) medium (Merck, Germany) overnight and adjusted to 1.5 × 10^8^ CFU/mL, then were diluted 1:100 and inoculated into a 96-well microtiter plate. Each isolate was investigated three times. The un-inoculated LB medium was used as a negative control. Following incubation of the microplate, the wells' contents were discharged and flushed with saline solution. Wells were stained with 0.1% crystal violet (Sigma–Aldrich, St Louis, USA). The crystal violet was aspirated and it’s remaining in the wells was solubilized by adding 95% ethanol (Flucka, Germany). The optical density (OD) of wells was measured at 570 nm and biofilm formation was assayed.

### Polymerase chain reaction (PCR) detection of resistance genes

DNA extraction was done from the *P. aeruginosa* isolates by the salting out method [25]. PCR assay was conducted on all the extracted DNA of the isolates using specific primers (Table 1). Electrophoresis detected amplicons on a 1% agarose (CinnaGen, Iran) gel in TBE (Tris–borate-EDTA) Buffer (CinnaGen, Iran). A 50 bp DNA ladder (MBI Fermentas, France) was utilized for comparisons. A representative for each positive PCR result was sequenced using the Applied Biosystems 3500.

**Table 1:** Primer sequences used for genes amplification by PCR

Genes	Primer Sequences (5’-3’)	Annealing temperature (°C)	Product Size (bp)	Refrences
*bla*_OXA-48_	F-GCGTGGTTAAGGATGAACAC R-CATCAAGTTCAACCCAACCG	58	438	[26]
*bla*_PER_	F-AATTTGGGCTTAGGGCAGAA R-ATGAATGTCATTATAAAAGC	56	925	[27]
*bla*_VEB_	F-CGACTTCCATTTCCCGATGC R-GGACTCTGCAACAAATACGC	55	643	[28]
bla_CTX-M_	F-TCTTCCAGAATAAGGAATCCC R-CCGTTTCCGCTATTACAAAC	55	909	[29]
*bla*_TEM_	F-TTTCGTGTCGCCCTTATTCC R-ATCGTTGTCAGAAGTAAGTTGG	60	403	[30]
*bla*_SHV_	F-TCAGCGAAAAACACCTTG R-TCCCGCAGATAAATCACC	52	472	[31]
*bla*_VIM_	F- AGTGGTGAGTATCCGACA R- ATGAAAGTGCGTGGAGAC	53	261	[27]
*bla*_GIM_	F- TCGACACACCTTGGTCTGAA R- AACTTCCAACTTTGCCATGC	52	477	[32]
*bla*_IMP_	F-ACCGCAGCAGAGTCTTTGCC R-ACAACAAGTTTTGCCTTACC	55	587	[27]
*bla*_GES_	F- ATGCGCTTCATTCACGCAC R- CAAAATTTTAAGACGGATCG	55	864	[27]
*bla*_SPM_	F-AAAATCTGGGTACGCAAACG R-ACATTATCCGCTGGAACAGG	58	271	[33]
*bla*_NDM-1_	F-GGTTTGGCGATCTGGTTTTC R-CGGAATGGCTCATCACGATC	52	621	[34]
*bla*_MCR-1_	F- CGGTCAGTCCGTTTGTTC R- CTTGGTCGGTCTGTAGGG	50	309	[35]

### Genotyping

The PCR method based on MLVA was conducted to amplify the VNTRs in the bacterial genome to determine various variants of *P. aeruginosa* isolates. The VNTR regions (Table 2) were chosen according to Vu-Thien et al. suggestion [36]. PCR products were dissociated in 1% agarose gel (CinnaGen, Iran). A 50 bp ladder (MBI Fermentas, France) was employed to determine the size of the amplicons. The size of the amplicons was analyzed by gel analyzer software [37]. To analyze the clusters, the Unweighted Pair Group Method with Arithmetic (UPGMA) technique was used with the BioNumerics 7.1 software (Applied Maths, Belgium). In addition, calculating the similarity coefficient of Pearson's correlation and the minimum spanning tree (MST) was implemented in BioNumerics 7.1 software (Applied Maths, Belgium). The dissimilarity of the isolates in conforming to the UPGMA algorithm was shown in the dendrogram. The Hunter-Gaston diversity index (HGDI) was utilized to assess the individual or combined VNTR loci polymorphism index. Observing one difference at any VNTRs was considered as a new genotype number. The clustering analysis using the categorical coefficient correlates with an interval of 85 to 100% similarity.

**Table 2:** Primer sequences used for multiple VNTRs loci in the PCR reactions

Locus name	Primer name	Primer Sequences (5’-3’)	Repeat unit size (bp)	HGDI index^a^
ms142	ms142L ms142R	AGCAGTGCCAGTTGATGTTG GTGGGGCGAAGGAGTGAG	115	0.81
ms211	ms111L ms111R	ACAAGCGCCAGCCGAACCTGT CTTCGAACAGGTGCTGACCGC	101	0.76
ms212	ms112L ms112R	TGCTGGTCGACTACTTCGGCAA ACTACGAGAACGACCCGGTGTT	40	0.75
ms213	ms113L ms113R	CTGGGCAAGTGTTGGTGGATC TGGCGTACTCCGAGCTGATG	103	0.85
ms214	ms114L ms114R	AAACGCTGTTCGCCAACCTCTA CCATCATCCTCCTACTGGGTT	115	0.81
ms215	ms115L ms115R	GACGAAACCCGTCGCGAACA CTGTACAACGCCGAGCCGTA	129	0.80
ms216	ms116L ms116R	ACTACTACGTCGAACACGCCA GATCGAAGACAAGAACCTCG	113	0.64
ms217	ms117L ms117R	TTCTGGCTGTCGCGACTGAT GAACAGCGTCTTTTCCTCGC	109	0.79
ms222	ms122L ms122R	AGAGGTGCTTAACGACGGAT TGCAGTTCTGCGAGGAAGGCG	101	0.76
ms223	ms123L ms123R	TTGGCAATATGCCGGTTCGC TGAGCTGATCGCCTACTGG	106	0.77

^a^Hunter-Gaston discriminatory index

### Statistical analyses

To analysis the data of this study, descriptive statistics (frequency and percentage) were used, and for this purpose Statistical Package for the Social Sciences (SPSS) version 16 was utilized. ​BioNumerics 7.1 software was used to analyze MLVA results.

## Results

Multidrug resistance *P. aeruginosa* has increased worldwide, which is considered a threat to public health. Our study investigated the phenotypic and genotypic antimicrobial resistance and molecular typing of *P. aeruginosa* isolates from Coronavirus disease-19 patients. Overall, 15 clinical isolates of *P. aeruginosa* were collected from blood cultures and endotracheal aspirates of COVID-19 patients in the ICU. The hypertension (60%), diabetes mellitus (33.3%), and ischemic heart disease (33.3%) were the most prevalent comorbidities. Six (40%) of the patients did not have any underlying disease. Table 3 showed the demographic characteristics of the patients hospitalized in ICU and infected with *P. aeruginosa* in the COVID-19 pandemic waves in Iran.

**Table 3:** Demographic characteristics of patients and the frequency of *P. aeruginosa* isolated from COVID-19 pandemic waves

Characteristics	*P. aeruginosa* isolates No (%)
**Age groups**	
15–25	0(0)
26–35	1(6.67)
36–45	0(0)
46–55	3(20)
56–65	4(26.67)
66–75	5(33.33)
76–85	0(0)
86–95	1(6.67)
96–105	1(6.67)
**Gender**	
Male	10(66.67)
Female	5(33.33)
**COVID-19 pandemic waves in Iran**	
Third (November, 2020)	5(33.33)
Fourth (April, 2021)	4(26.67)
Fifth (August, 2021)	6(40)
**Underlining disease**	
0	6(40)
1	2(13.33)
1, 2	2(13.33)
1, 2, 3	3(20)
1, 3	1(6.67)
1,3, 4	1(6.67)
**Fate**	
Deceased	7(46.67)
Discharged	8(53.33)

0: No underlying disease; 1: Hypertension; 2: Diabetes; 3: Heart failure; 4: Brain aneurysm

### Phenotypic characteristics of *P. aeruginosa* isolates

Using the biochemical tests, all 15 isolates were confirmed as *P. aeruginosa.*

### Antimicrobial susceptibility testing

The antimicrobial susceptibility of *P. aeruginosa* isolates were reported as follows: 14(93.3%) isolates resistant to imipenem, 14(93.3%) to co-trimaxazole, 12(80%) to ceftriaxone, 12(80%) to ceftazidime, 9(60%) to gentamicin, 9(60%) to levofloxacin, 9(60%) to ciprofloxacin, and 9(60%) isolates resistant to cefepime. Ten (66.6%) isolates were identified as MDR (Table 4). In the MIC method, 15(100%), 15(100%), 3(20%), and 2(13.3%) *P. aeruginosa* isolates were resistant to imipenem, meropenem, polymyxin B, and colistin, respectively.

**Table 4:** Antimicrobial categories and agents used to define MDR, XDR and PDR* P. aeruginosa isolates*

Antimicrobial category	Antimicrobial agent	No (%) of resistant isolates	Type of resistance No (%)		antibiotic-resistance genes					
					No (%) of positive ESBL isolates		No (%) of positive Carbapenemase isolates		No (%) of Colistin resistance isolates	
Aminoglycosides	Gentamicin	9 (60)	Non-MDR	5 (33.3)	P	EG^a^	P	CG^b^	P	MG
Carbapenems	Imipenem	14 (93.3)								
Cephalosporins	Ceftazidime	12 (80)	MDR	1 (6.6)	3 (20)	3 (100)	10 (66.6)	10 (100)	2 (13.3)	0
	Cefepime	9 (60)								
Fluoroquinolones	Ciprofloxacin	9 (60)								
	Levofloxacin	9 (60)	MDR, possible XDR	8 (53.3)						
Penicillins + β-lactamase inhibitors	NT	-								
Monobactams	NT	-								
Phosphonic acids	NT	-	MDR, possible XDR, possible PDR	1 (6.6)						
Polymyxins	Colistin	2 (13.3)								
	Polymyxin B	3 (20)								

*NT* not tested for susceptibility to antimicrobial agent in this category, *P* Phenotypic, *EG* ESBL genes (Positive for at least one gene in phenotypically positive isolates), *CG* Carbapenemase genes (Positive for at least one gene in phenotypically positive isolates)

^a^ESBL genes (*bla*_TEM_, *bla*_SHV_, *bla*_PER_, *bla*_CTX-M_, and *bla*_VEB_)

^b^Carbapenemase genes (*bla*_OXA-48_, *bla*_IMP_, *bla*_SPM_, *bla*_VIM_, *bla*_GIM_, *bla*_GES_, and *bla*_NDM_); MG: Colistin resistance *gen (bla*_MCR-1_)

MDR: non-susceptible to ≥ 1 agent in ≥ 3 antimicrobial categories

XDR: non-susceptible to ≥ 1 agent in all but ≤ 2 categories

PDR: non-susceptible to all antimicrobial agents listed

### ESBLs and carbapenemases producing *P. aeruginosa*

**Fig. 1:**
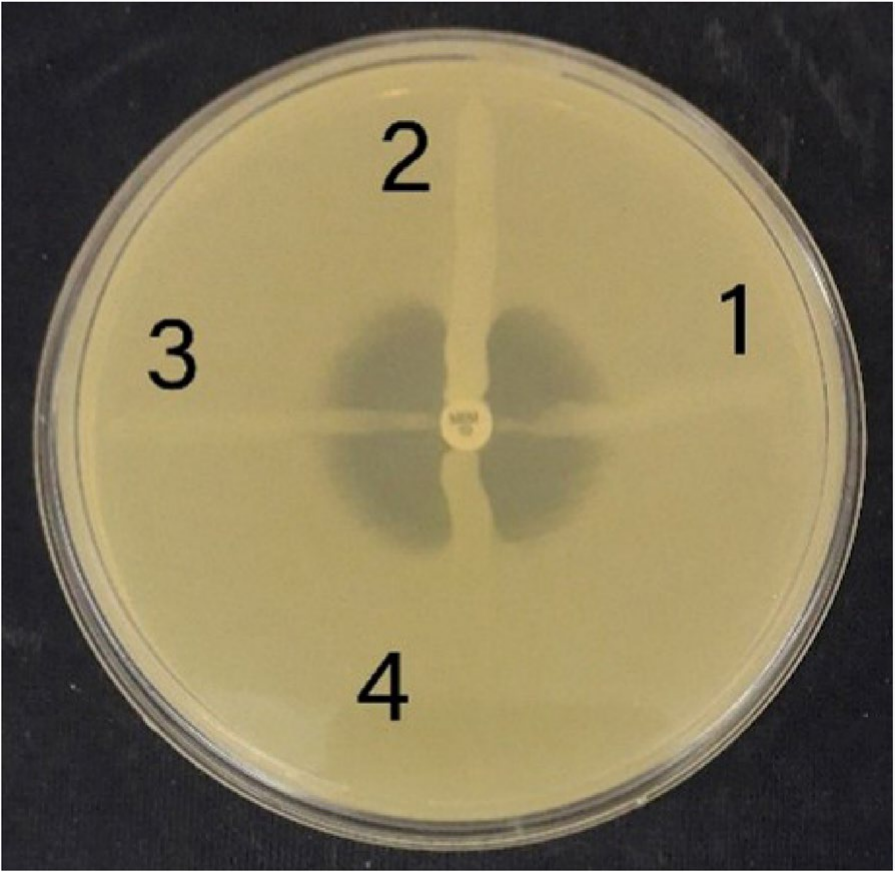
Modified Hodge test; isolates 2 and 4 show positive results, isolates 1 and 3 show negative results

Detection of carbapenemases by MHT demonstrated that 10(66.6%) *P. aeruginosa* isolate were positive (Fig. 1) and ESBLs were identified in 3(20%) *P. aeruginosa* isolates.

### Biofilm formation and quantification

The results demonstrate that 15(100%) *P. aeruginosa* isolates were positive for biofilm formation, which 11(73.3%) and 4(26.7%) isolates were strong and moderate biofilm producers, respectively.

### PCR results

The results of PCR for ESBLs *bla*_TEM_, *bla*_PER_, *bla*_SHV_, and *bla*_CTX-M_ genes were detected in 13(86.6%), 3(20%), 1(6.6%), and 1(6.6%) of the isolates, respectively. The *bla*_*VIM*_*, bla*_*GIM*_*, bla*_*GES*_*, and bla*_*MCR-1*_ genes were not identified in any of the isolates. The *bla*_OXA-48_, *bla*_IMP_, *bla*_SPM_, *bla*_VEB_, and *bla*_NDM_ were detected in 15(100%), 13(86.6%), 6(40%), 3(20%), and 2(13.3%) of the isolates, respectively. Eleven (73.3%) isolates carried *bla*_*OXA-48*_*, bla*_*IMP*_*,* and *bla*_*TEM*_ genes, and 6 (40%) isolates also carried *bla*_*OXA-48*_*, bla*_*TEM*_*,* and *bla*_*SPM*_ genes, simultaneously.

### MLVA typing

**Fig. 2:**
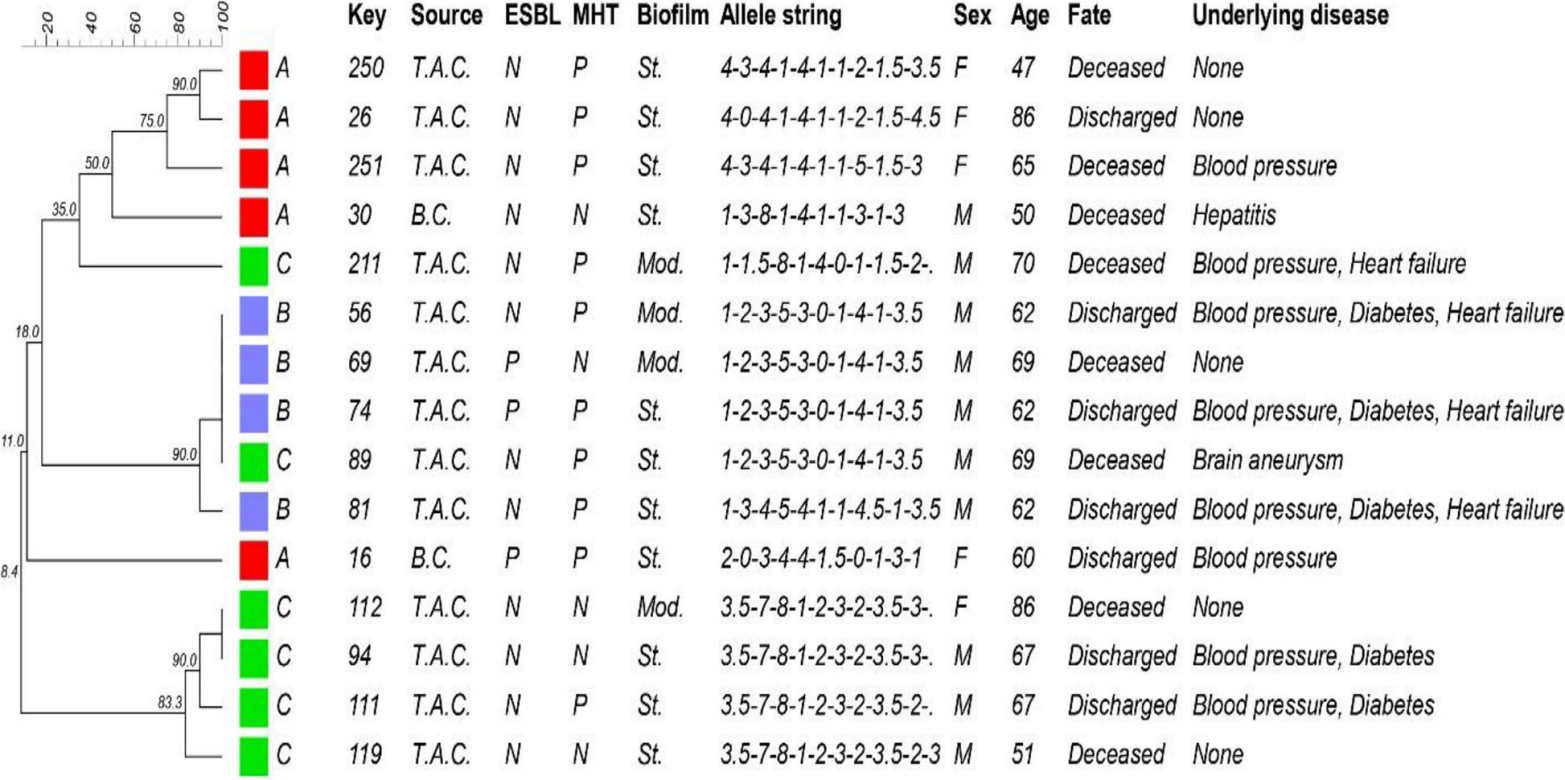
Inferred dendrogram from the clustering analysis of MLVA results of 15 *P. aeruginosa* isolates using the UPGMA algorithm. A: third wave of COVID-19, B: fourth wave of COVID-19, C: fifth wave of COVID-19, Key: isolates ID, T.A.C: tracheal aspirates culture, B.C: blood culture, ESBL: Extended-spectrum-β-lactamases, MHT: Modified Hodge Test, N: negative, P: positive, St: strong biofilm producer, Mod: moderate biofilm producer, F: female, M: male, None: No underlying disease

**Fig. 3:**
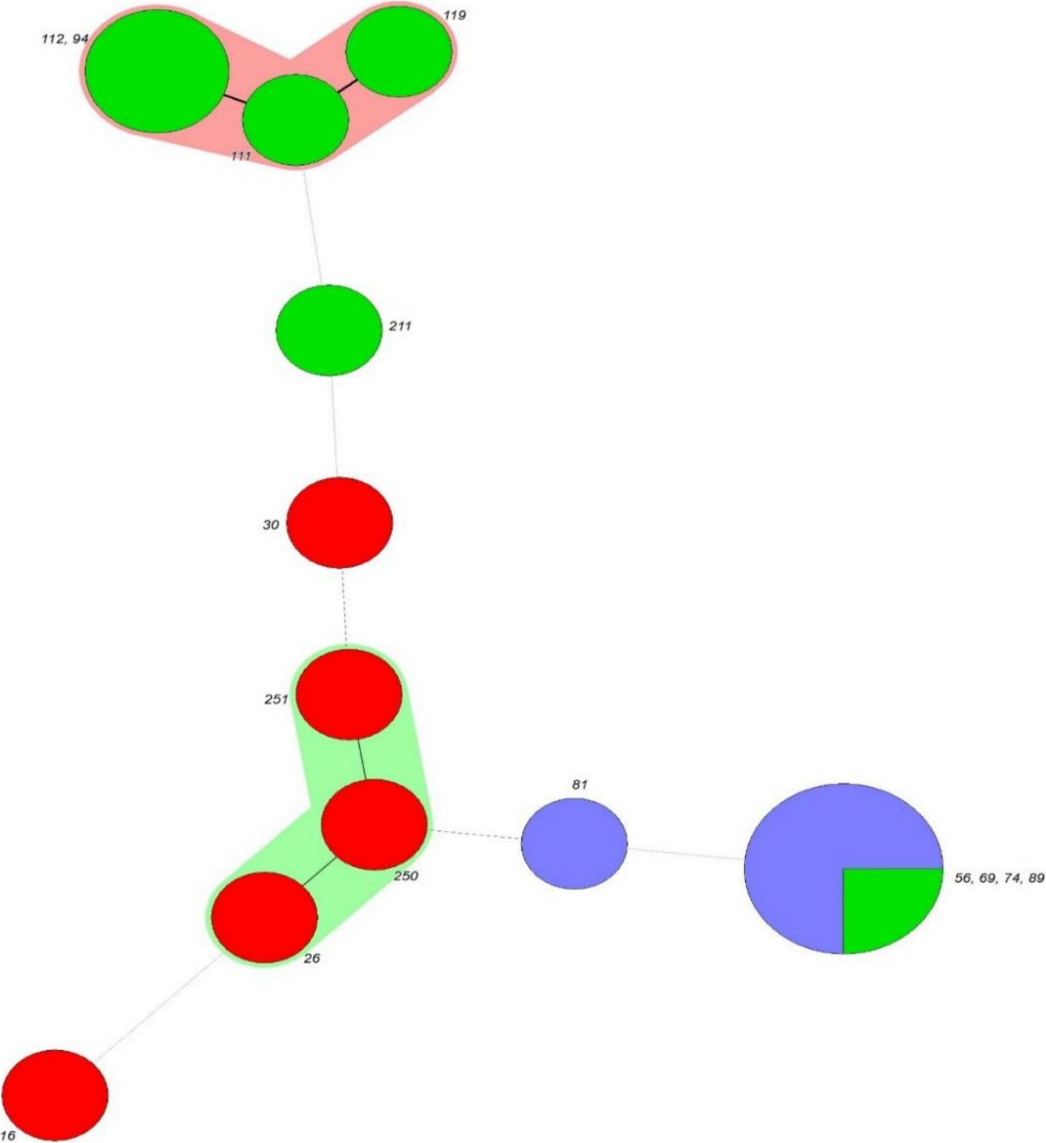
Minimum Spanning Tree (MST) algorithm obtained from the 15 *P. aeruginosa* isolates by MLVA. Each circle indicates a single type, the size showing the number of isolates with this specific type. The numbers on a single circle are 100% identical to each other. Thick black lines connecting pairs of MLVA-types display that they differ in one VNTR locus, thin black lines connecting pairs of MLVA-types show that they differ in two VNTR loci, and dashed lines connecting pairs of MLVA-types show that they differ in three VNTR loci. Pink and light green zones surround MLVA-types that belong to the same MLVA clonal complexes (MLVA-CC)

The molecular typing of 15 clinical isolates of *P. aeruginosa* were evaluated by the MLVA method regarding the amplification of ten different VNTR regions. Generally, there were 11 different MLVA types of *P. aeruginosa*, which the most frequent types belonged to types 6 with 4 isolates, and type 9 with 2 isolates. Eleven various MLVA types of *P. aeruginosa* isolates were allocated to seven clusters (Fig. 2). The MST algorithm originated from the MLVA genotyping for the clinical isolates of *P. aeruginosa* shown in Fig. 3.

## Discussion

COVID-19 patients are particularly prone to superinfection and secondary bacterial infections. However, the pattern of bacterial co-infections associated with COVID-19 and the microbiological profile in these cases are not widely studied [38]. Therefore, it is essential to monitor bacterial co-infection in COVID-19 patients, especially with multi-drug resistant bacteria, to control hospital infections. Antibiotic resistance is a serious crisis that threatens global health and needs urgent action. In prior investigations, resistance to carbapenem varied significantly from 17.5% to 100% [39–41]. A recent study reported that *P. aeruginosa* isolated from COVID-19 patients showed 100% resistance to ciprofloxacin, levofloxacin, co-trimaxazole, cefotaxime, cefepime, meropenem, and imipenem and 50% resistance to gentamicin [42]. In a recent study done in Iraq by Tizkam et al., *P. aeruginosa* isolated from COVID-19 patients showed 100% resistance to ceftriaxone and gentamicin, 48.9% and 50% resistance to levofloxacin and meropenem, respectively [43]. Based on Jamnani et al. study, *P.aeruginosa* isolated from ICU-admitted COVID-19 patients was 100% resistant to cefixime and co-trimaxazole, 50% resistant to ciprofloxacin, 25% resistant to gentamycin and colistin [44]. In this study, colistin (86.6%) showed the highest rate of susceptibility, similar to prior investigations [45, 46]. It can be attributed to factors such as the high cost of colistin and its limited use outside hospitals. Discrepancies in antibiotic susceptibility patterns between isolates in various countries can be explained by the source of isolates, the rise of empiric antibiotic use, the existence or lack of antibiotic use supervision schedules, horizontal gene transfer, and discrepancies in the region's epidemiology.

Multiple mechanisms of resistance to antibiotics exist in bacteria, including reduced permeability, expression of efflux pumps, generation of antibiotic-inactivating enzymes, and target modifications. Most of these resistance mechanisms are present in *P. aeruginosa*. By producing these mechanisms simultaneously, MDR, XDR, and PDR strains emerge [47]. A recent study showed the most abundant species of bacteria isolated from severe COVID-19 patients were *P. aeruginosa* (39.5%), and 64.7% of these isolates also were multi-drug-resistant strains [48]. Another study detected MDR isolates in 64.5% of COVID-19 patients [49]. Typical resistance mechanisms in pathogens isolated from COVID-19 patients have rarely been examined. Similarly, low production (27.3%) of ESBL was reported by Dutta et al., while Farhan et al. study presented ESBL production in 54% MDR P. aeruginosa [50, 51]. In the other study, the production rate of ESBL and carbapenemase enzymes was stated at 24.7% and 25.7% in *P.aeruginosa* isolates, respectively [52]. The emergence and rapid spread of β-lactamase enzymes producing bacteria are of serious concern and threat. Thus, it is essential to examine β-lactamase enzymes producing isolates.

Hence, for COVID-19 subjects that exhibit co-infections with other respiratory infections, the immediate administration of antimicrobial agents relevant to the Antibiotic sensitivity test results and also accurate application of infection control protocols are required to alleviate mortality and hospital spread [53–55]. Moreover, the isolates were assessed for the presence of several resistance genes. A recent report indicated that the gram-negative clinical isolates from COVID-19 Patients were mostly multidrug-resistant and ESBL and/or carbapenemase producers and carried different resistance-associated genes, including *bla*_NDM-1_, *bla*_TEM_, *bla*_CTX-M_, and *bla*_SHV_. According to their results, 100% of *P. aeruginosa* isolates carried *bla*_NDM-1_ and *bla*_TEM_, and no isolates had *bla*_CTX-M_ and *bla*_SHV_ [56]. In contrast to our study, in Farhan et al. Study, *bla*_CTX-M15_ was detected in 55.5% positive ESBL *P. aeruginosa*, and *bla*_IMP_, *bla*_VIM_, and *bla*_GIM_ were found in 42.8%, 52.3%, and 52.3% of carbapenem-resistant *P. aeruginosa*, respectively. In a study in Iraq, out of 20 *P. aeruginosa* isolates isolated from Covid-19 patients hospitalized in ICU, 16 (80%) isolates were positive for *bla*_*CTX-M*_*,*but *bla*_*SHV*_* and bla*_*TEM*_ were not found in any of the tested isolates [57]. Furthermore, Similar to our findings, reported for incidence of *bla*_SPM_ (38%) among *P. aeruginosa* isolates [51]. Ahmed et al. reprted a low prevalence of positive *bla*_CTX-M_ *P. aeruginosa* isolates(10.7%), which is consistent with the results of our study [58]. In contrast to current study, Tawfik et al. reported 68% and 20% the prevalence of *bla*_VEB_, and *bla*_GES_ in *P. aeruginosa*, respectively. Also, ESBLs *bla*_TEM_, *bla*_SHV_, *bla*_PER_, and *bla*_CTX-M_ genes were not reported [59]. Based on the findings of Bianco et al., among the 1242 clinical isolates of Enterobacterales during the Covid-19 epidemic, 1034 (83.2%), 114 (9.2%), 53 (4.3%) and 51 (4.1%) isolates were positive for *KPC, bla*_VIM_, *bla*_OXA-48_ and *bla*_NDM_, respectively [60]. According to Miftode et al. Study, 72 (82.7%) of the 87 Enterobacterales isolated from covid-19 patients produced carbapenemases and 26 (36.1%), 25 (34.7%), 13 (18%), and 2 (2.7%) isolates were positive for *bla*_*OXA-48*_*, bla*_*NDM*_*, KPC, and bla*_*VIM*_*,* respectively [61]. However, resistance genes rate vary significantly between various studies, which can lead to diversity in infection management guidelines. Prior studies showed that viral infections, including COVID-19, promote bacterial biofilm formation [62–64]. In recent studies, 92.7% and 94% *P. aeruginosa* isolates were reported as biofilm producers [39, 65]. Typing methods have a substantial role in comprehending the epidemiology relevant to severe nosocomial infections caused by *P. aeruginosa* [66]. Detection of diverse strains of various bacterial types is necessary for the investigation of the prevalence and control of bacterial infections [67]. Regarding the great genetic variety of MDR *P. aeruginosa* isolates, especially seen in ICU, implementing appropriate infection management procedures is challenging. In the present study, the amplification of the ten distinct VNTR loci showed all studied *P. aeruginosa* isolates were typeable. In contrast, Lalancette et al. study reported three not-typable strains [68]. Regarding observed high genetic diversity among *P. aeruginosa* isolates, separated from COVID-19 patients, it is essential the continuous monitoring of the molecular epidemiology of *P. aeruginosa* isolates in the COVID-19 epidemic. The current study has some limitations. We have no information about COVID-19 patients who did not develop co-infections (control patients), the type and number of antimicrobials prescribed during the COVID-19 pandemic, and the length of hospitalization. We suggest that subsequent work comprise such information to allow comparative analysis.

## Conclusions

At the beginning of the Covid-19 pandemic, the existing guidelines for COVID-19 patients did not include specific recommendations for the use of antibiotics or specific management measures to prevent nosocomial infections in these patients. Due to the excessive use of antibiotics during the pandemic, there was a significant increase in antibiotic resistance. It was worried that the existent overuse of antimicrobial agents during the COVID-19 pandemic could accelerate the emersion of the subsequent global public health crisis caused by the resistance of microorganisms to a variety of drugs. Due to the high rate of antimicrobial resistance, as well as the genetic diversity of *Pseudomonas aeruginosa* isolates from COVID-19 patients, the current study emphasizes the significance of monitoring local epidemiology, which might be helpful in antimicrobial agents use and surveillance programs.

## Data Availability

Data is available from the corresponding author upon request.

## Abbreviations

COVID-19: Coronavirus disease 2019
MLVA: Multilocus variable number tandem repeat analysis
ICU: Intensive care unit
MDR: Multidrug-resistant
SARS-CoV-2: Severe acute respiratory syndrome coronavirus 2
VNTRs: Variable number of tandem repeats
ESBLs: Extended-spectrum-β-lactamases
CLSI: Clinical and Laboratory Standards Institute
MH: Mueller–Hinton
MIC: Minimum inhibitory concentration
CDT: Combination disk test
MHT: Modified Hodge test
LB: Luria–Bertani broth
MTP: Microtiter plates
HGDI: Hunter-Gaston discriminatory index
UPGMA: Unweighted Pair Group Method with Arithmetic
MST: Minimum spanning tree
